# Fibroblast involvement in soft connective tissue calcification

**DOI:** 10.3389/fgene.2013.00022

**Published:** 2013-03-05

**Authors:** Ivonne Ronchetti, Federica Boraldi, Giulia Annovi, Paolo Cianciulli, Daniela Quaglino

**Affiliations:** ^1^PXELab, University of Modena and Reggio EmiliaModena, Italy; ^2^Department of Life Science, University of Modena and Reggio EmiliaModena, Italy; ^3^U.O.D.S Unit, S. Eugenio HospitalRome, Italy

**Keywords:** fibroblasts, PXE, PXE-like disorders, elastin, extracellular matrix, ectopic calcification, mesenchymal stromal cells

## Abstract

Soft connective tissue calcification is not a passive process, but the consequence of metabolic changes of local mesenchymal cells that, depending on both genetic and environmental factors, alter the balance between pro- and anti-calcifying pathways. While the role of smooth muscle cells and pericytes in ectopic calcifications has been widely investigated, the involvement of fibroblasts is still elusive. Fibroblasts isolated from the dermis of pseudoxanthoma elasticum (PXE) patients and of patients exhibiting PXE-like clinical and histopathological findings offer an attractive model to investigate the mechanisms leading to the precipitation of mineral deposits within elastic fibers and to explore the influence of the genetic background and of the extracellular environment on fibroblast-associated calcifications, thus improving the knowledge on the role of mesenchymal cells on pathologic mineralization.

## CALCIFICATIONS IN SOFT CONNECTIVE TISSUES

For long time, unwanted calcification, as that occurring in arterial calcification and in nephrolithiasis, has been considered as a passive, physical–chemical phenomenon representing a degenerative, irreversible process often associated with aging (Shroff and Shanahan, 2007). Many recent investigations, however, pointed out that calcium and phosphate precipitation are the result of complex and highly regulated series of events in which the balance between calcification inducers and inhibitory mechanisms may become severely deranged locally and/or systemically.

The deposition of calcium and phosphate in soft connective tissues can be classified into three major categories: metastatic calcification, dystrophic calcification, and calcinosis (Black and Kanat, 1985). Metastatic calcification occurs when calcium–phosphorous levels are elevated mainly due to metabolic/hormonal alterations and/or to tumor-associated complications. Dystrophic calcification takes place in the presence of damaged or necrotic tissue as in atherosclerosis. Calcinosis is generally associated to hypovascularity or hypoxia, it may involve a localized area or it may be widespread, causing secondary muscle atrophy, joint contractures and skin ulceration, with recurrent episodes of inflammation or infection (Boulman et al., 2005).

In most cases, mineral deposition develops in the extracellular environment without being localized on specific matrix components/structures. A typical example is represented by “calciphylaxis,” a rare disease in which a generalized calcification is associated with thrombotic cutaneous ischemia and necrosis, thus causing a mortality rate ranging from 60 to 80% due to wound infection, sepsis, and subsequent organ failure (Arseculeratne et al., 2006; Hoff and Homey, 2011).

As clearly shown by several experimental findings and clinical observations, calcification may also occur in a number of genetic diseases, in metabolic disorders, such as uremia, hyper-parathyroidism, and diabetes, or in areas without adjacent inflammation or atherosclerosis. Due to the heterogeneity of factors contributing to the development of calcifications, many studies have been carried out in order to find common pathogenetic mechanisms and to identify possible druggable targets (i.e., single molecules and/or signaling pathways). Within this framework, numerous proteins have been identified to be involved in bone calcification as well as in ectopic mineralization. It has been suggested that an active and dynamic balance of pro- and anti-calcifying mechanisms occurs in both physiological and pathological calcification (Abedin et al., 2004) and that mesenchymal cells are key players, not only because they synthesize most of the mineral regulatory proteins, but also because they are responsible for the qualitative and quantitative characteristics of the extracellular environment, where apatite ectopic deposition arises.

### ROLE OF PRO- AND ANTI-CALCIFYING FACTORS IN ECTOPIC CALCIFICATION

**FIGURE 1:**
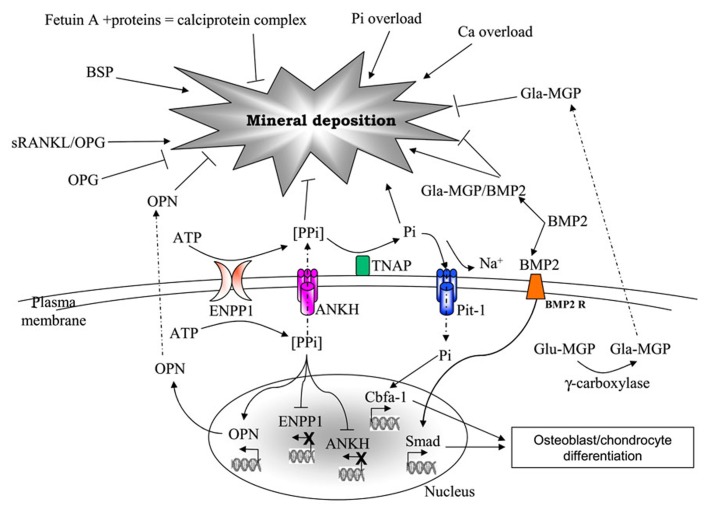
**Major factors involved in mineral deposition**. AMP, adesosine monophosphate; ANKH, ankylosis protein homolog; ATP, adenosine triphosphate; BMP2, bone morphogenetic protein-2; BMP2R, bone morphogenetic protein-2 receptor; BSP, bone sialoprotein; Ca, calcium; ENPP1, ectonucleotide pyrophosphatase/phosphodiesterase; Glu- and Gla-MGP, uncarboxylated- and carboxylated-matrix Gla protein; OPG, osteoprotegerin; OPN, osteopontin; Pi, inorganic phosphate; Pit-1, phosphate transporter-1; PPi, pyrophosphate; RANKL, receptor activator of nuclear factor kappa-B ligand; TNAP, tissue non-specific alkaline phosphatase.

The role of calcitropic hormones, namely catecholamines, parathyroid hormone (PTH), and vitamin D or 1,25(OH)_2_D_3_ on calcium metabolism is well-known (Rizzoli and Bonjour, 1998). However, in the last decade, a growing number of evidence is highlighting the importance of many other molecules as part of a composite network that, on the basis of common structural components, exhibits peculiar interactions and/or undergoes different regulatory mechanisms depending on the tissue [e.g., osteoprotegerin (OPG) or matrix Gla protein (MGP) in bone and vascular tissue; Kornak, 2011] and on the environmental context. In addition, these molecules can be produced and locally secreted by mesenchymal cells, or can diffuse from circulation to peripheral tissues, where they may exert different effects on local calcium/phosphate homeostasis (**Figure 1**).

The mechanisms of calcification in skeletal and dental tissues have been under investigation since long time. One common feature to almost all physiological mineralization mechanisms seems the involvement of small (20–200 nm) membrane particles, called matrix vesicles (MVs). They bud off from the plasma membrane of mineralizing cells and are released into the pre-mineralized organic matrix serving as a vehicle for the concentration of ion or ion-enriched substrates, which are required for the activity of membrane-bound enzymes triggering mineral deposition at specific sites.

The observation that MV-like membranes are present in a number of ectopic calcification processes supports the concept that the mechanisms of vascular calcification are similar to those seen in normal skeletal development (Golub, 2011).

However, soft connective tissue calcifications activate a number of common pathways, but, at the same time they may exhibit local specific variations (e.g., in different tissue/body regions), possibly depending on the genotypic/phenotypic peculiarities of each mesenchymal cell type/subtype. Mineralization of dermal constituents, for instance, has been never associated with MVs, indicating that fibroblasts, differently from smooth muscle cells, can be responsible for mineral deposition, even in the absence of MVs. It could be, therefore, hypothesized that the role of mesenchymal cells in ectopic calcification may differ depending on the ability of the cell type to acquire a bone-oriented phenotype.

To further increase the complexity and the heterogeneity of mechanisms regulating pathologic calcification there are studies demonstrating that factors promoting or inhibiting ectopic calcifications are under the control of different genes, as in the case of extracellular pyrophosphate (PPi), a small molecule made of two phosphate ions, linked by an ester bond, that regulates cell differentiation and serves as an essential physiologic inhibitor of calcification by negatively interfering with crystal growth (Terkeltaub, 2001). The amount of extracellular PPi is regulated by two different gene products, as it originates either from the breakdown of nucleotide triphosphates by the ectonucleotide pyrophosphatase/phosphodiesterase (PC-1/ENPP1) or from the PPi transport by the transmembrane ankylosis protein homolog (ANKH). Consistently, either mutations or knockdown of these genes can induce hyper-mineralization of aorta (i.e., generalized arterial calcification in infants or GACI) and of ligaments and articular cartilage (i.e., chondrocalcinosis) in humans and mice, respectively (Okawa et al., 1998; Ho et al., 2000; Pendleton et al., 2002; Rutsch et al., 2003).

In the extracellular space, phosphate levels are directly controlled by tissue non-specific alkaline phosphatase (TNAP), a cell membrane-bound ecto-enzyme that increases inorganic phosphate availability by releasing it from a variety of phosphate-enriched substrates and, at the same time, reduces the levels of calcification inhibitors, promoting the hydrolysis of PPi and the dephosphorylation of osteopontin (OPN; El-Abbadi et al., 2009; Orimo, 2010). As a consequence, expression and activity of TNAP are associated either with physiological and pathological calcifications, although changes in enzyme activity may not be directly proportional to the level of mineralization, which is actually the result of the activity of many genes/proteins (Mendes et al., 2004). Consistently, increased TNAP expression and activity have been observed in CD73 deficiency, a disorder that, due to mutations in NT5E (a gene encoding for the membrane-bound ecto-enzyme that cleaves adesosine monophosphate (AMP) to adenosine and inorganic phosphate), is characterized by tortuosity and calcification of lower limb arteries and by mineralization of hand and foot joint capsules (StHilaire et al., 2011).

Alkaline phosphatase, similarly to other osteogenic genes, as type I collagen, osteocalcin (OC), and bone sialoprotein (BSP), can be transcriptionally regulated by bone morphogenetic protein 2 (BMP2; Kim et al., 2004), a powerful cytokine that, by activating Smad signaling pathways, promotes differentiation of mesenchymal cells into osteoblasts *in vitro* and induces bone formation *in vivo* (Rosen, 2009). Consistently, in fibrodysplasia ossificans progressiva endochondral ossification is triggered by BMP signaling in muscle cells (Shen et al., 2009). It has been demonstrated that treatment of smooth muscle and bone cell cultures with BMP2 (i) promotes osteogenic phenotype transition of smooth muscle cell (SMC; i.e., up-regulation of Runx2/Cbfa1 and down-regulation of SM22 expression), (ii) enhances elevated phosphate-induced calcification, but does not induce calcification under normal phosphate conditions. These results clearly indicate that phosphate transport via Pit-1 is crucial in BMP2-mediated calcification and in cell phenotype modulation (Suzuki et al., 2006; Li et al., 2008). Pit-1 is a type III sodium-dependent phosphate co-transporter that, through the activation of the Erk 1/2 signaling pathways, promotes calcification and favors changes of vascular smooth muscle cell (VSMC) toward an osteochondrogenic phenotype. Moreover, it has been shown that Pit-1 may exert effects also at the endoplasmic reticulum level. Studies on VSMC revealed that, when these cells are treated with platelet-derived growth factor (PDGF), they exhibit increased Pit-1 expression and it has been hypothesized that Pit-1 may regulate anti-calcification proteins (such as MGP), as well as kinases able to phosphorylate secreted matrix proteins (such as OPN; Villa-Bellosta et al., 2007). In addition, recent evidence has been provided that Pit-1 have other unexpected functions in cell proliferation and embryonic development (Lau et al., 2010), thus emphasizing the regulatory importance of phosphate in cell behavior.

Another protein favoring calcification is BSP, originally identified in bone and at sites of ectopic calcification in blood vessels, heart valves, and skeletal muscle. It is involved in the early stages of mineralization and bone desorption, since it is immobilized on collagen fibrils where the poly-glutamic acid sequences of BSP act as possible nucleation sites for hydroxyapatite crystals. BSP, together with another bone phosphoprotein named OPN, can modulate crystal shape by adsorption on a specific face of the crystals (Ganss et al., 1999).

Osteopontin is in fact a highly phosphorylated and glycosylated secreted protein originally discovered in bone, but identified also in calcified vascular lesions (Giachelli et al., 1993), where it may counteract apatite deposition by physically inhibiting crystal growth (Boskey et al., 1993) and/or by up-regulating the expression of genes, as carbonic anhydrase II, favoring mineral absorption, mainly through the activation of macrophage activities (Rajachar et al., 2009). These properties depend on the level of OPN phosphorylation as well as on the targeted tissue (i.e., bone or soft connective tissues; Jono et al., 2000). Recent evidence puts forward that OPN is actually a multi-functional protein able to interact with several integrin receptors, thus playing a role in activation, adhesion and migration of many cell types, not only in tissue mineralization and tumor growth, but also in inflammation (Jahnen-Dechent et al., 2008). These broad biological activities underlie the presumed role of OPN in the pathogenesis of cardiovascular diseases, including atherosclerosis and abdominal aortic aneurysm (Giachelli and Steitz, 2000), thus paving the way toward the clinical use of OPN plasma levels as biomarker of inflammation and as predictor of the risk for cardiovascular complications (Cho et al., 2009).

Another interesting protein is OPG that serves as a decoy receptor for the receptor activator of nuclear factor κB-ligand (RANKL) and acts as an inhibitor of osteoclastogenesis and osteoclast activation by blocking RANK activation (Boyle et al., 2003; Van Campenhout and Golledge, 2009). As demonstrated in the KO animals, the absence of OPG is associated with osteoporosis as well as with calcifications of aorta and renal arteries (Bucay et al., 1998). Therefore, within the vasculature, OPG may exert a protective role toward ectopic calcification down-regulating alkaline phosphatase activity (Van Campenhout and Golledge, 2009). Consequently, serum OPG levels have been significantly associated with the presence of coronary artery disease (Jono et al., 2002), suggesting that OPG may represent a strong risk factor for mortality in dialysis patients (Morena et al., 2006).

Matrix Gla protein belongs to a large family of proteins whose maturation requires vitamin K-dependent carboxylation of glutamyl residues (Schurgers et al., 2007; O’Young et al., 2011). It is considered the most active anti-calcifying agent in vessels (Shanahan et al., 1998; Price et al., 2006), but it is actually produced by several cell types, among which VSMCs, osteoblasts, and fibroblasts (Davies et al., 2006; Park et al., 2006). The phenotype of *MGP*-/- mice is characterized by arterial calcification and by arterial-venous malformations (Yao et al., 2011), suggesting that MGP has roles in connective tissue development and homeostasis, as well as in preventing ectopic calcification. The corresponding human disorder is Keutel syndrome (Munroe et al., 1999), characterized by enhanced mineralization of the growth plate cartilage leading to reduced longitudinal growth and osteopenia, as well as calcification of the elastic lamellae in the arterial wall due to chondrocyte transformation of VSMCs. This aberrant cellular differentiation could be related to the ability of MGP to act as a regulator of BMP2 in a dose-dependent manner. Low levels of MGP relative to BMP2 may result in mild enhancement of BMP2 activity, whereas intermediate levels would inhibit and high levels strongly increase BMP2 activity (Zebboudj et al., 2002). These findings clearly demonstrate the complexity of the mechanisms regulating ectopic calcifications, which are dependent not only on the presence/absence of specific proteins and on their activity (due, for instance, to post-translational modifications as phosphorylation and carboxylation), but also on the ratio among different molecules.

A similar vitamin K-dependent carboxylated protein is OC that, synthesized by osteoblasts, is deposited into bones, where it controls the size and the speed of crystal formation and acts as a chemo-attractant for osteoclasts (Roach, 1994). Moreover, it is released into circulation, where it is also used as a biomarker of bone metabolism and vitamin K status. The increase of under-carboxylated OC (ucOC) levels in the aging population led to the hypothesis that vitamin K insufficiency might be related to the calcification paradox (namely age-dependent bone loss associated to vascular calcification), however, clinical trials did not provide support to the hypothesis that vitamin K supplementation will reduce bone loss or fracture risk. Very recent results from *in vitro* and *in vivo* experimental models indicate that ucOC is an active hormone with a positive role on glycemia. If this hypothesis will be proved also in humans, vitamin K supplementation, by decreasing ucOC, might exert unknown, possibly detrimental, effects on glucose metabolism (Gundberg et al., 2012). This hypothesis sustains the importance to perform broad and extended investigations when diet regimens, supplemented with even physiological/endogenous components, are used as therapeutic tools. Interference with a specific molecule may in fact have “domino” consequences on many other, apparently unrelated, pathways.

Finally, a novel γ-carboxyglutamate (Gla)-containing protein, named Gla-rich protein (GRP) due to its high content in Gla residues, has been identified in association with chondrocytes and bone cells. Although its molecular function is yet unknown, the high content of Gla residues and its accumulation at sites of pathological calcification in skin, vascular system and breast cancer tumors suggest that GRP modulates calcium availability, regulates cartilage matrix organization and influences matrix stability being associated with fibrillar collagens (Cancela et al., 2012).

Although not synthesized by mesenchymal cells, being secreted from hepatocytes into the circulation, never the less, fetuin A exerts its biological role in the periphery, where it inhibits calcification by the transient formation of soluble colloidal spheres (Heiss et al., 2003). It binds calcium phosphate and calcium carbonate with high affinity and, although with lower efficiency, magnesium phosphate (Schinke et al., 1996). In rat sera fetuin A is present in high molecular weight complexes, termed “calciprotein” particles, which contain calcium, phosphate and matrix Gla protein. They act as inhibitors of mineralization in solution and of cell-mediated mineralization by inhibiting the *de novo* formation of calcium phosphate without dissolving preformed minerals (Schlieper et al., 2007).

The complexity of the mechanisms regulating pathologic calcification is further highlighted by the involvement of apparently unrelated gene products, as it was noticed for Klotho and multi-drug resistance protein 6 (MRP6), just to mention few of them. Klotho is a transmembrane protein with an extracellular (β-glucosidase domain that can be shed from the plasma membrane by Adamts proteases and, in addition to its enzymatic function, binds directly to fibroblast growth factor (FGF)23 acting as an essential FGF-coreceptor. In the kidney, FGF23 signalling leads to a down-regulation of the sodium/phosphate co-transporter (NaPi) and of the vitamin D 1α-hydroxylase. Therefore, Klotho deficiency, in spite of high FGF23 levels and of high 1,25-dihydroxy vitamin D3 and calcium concentrations, leads to osteopenia, hyper-phosphatemia, and consequently widespread vascular and soft tissue calcifications (Moe, 2012). By contrast, dysfunction of the ATP-binding cassette (ABC)-transporter *ABCC6* (coding for the transmembrane protein MRP6 highly expressed in liver and kidney) causes pseudoxanthoma elasticum (PXE), a rare disease characterized by mineralization and degeneration of elastic fibers within soft connective tissues, thus causing skin laxity, cardiovascular complications, and visual impairment in a setting of normal levels of circulating calcium and phosphate and without bone abnormalities (see ahead for further details; Quaglino et al., 2011).

### ROLE OF THE EXTRACELLULAR MATRIX IN ECTOPIC CALCIFICATION

Changes in the characteristics of the extracellular matrix and in the ratio between matrix constituents influence not only the mechanical properties of connective tissues, but significantly contribute to modulate cell phenotype by altering integrin expression, focal adhesions, cytoskeletal organization and consequently intracellular signaling pathways.

As a consequence, it has been shown that osteogenic differentiation of calcifying VSMCs was promoted by type I collagen and fibronectin, but it was inhibited by type IV collagen. By contrast, valvular interstitial cells (a heterogeneous population of fibroblasts, with a small percentage of myofibroblasts and smooth muscle cells ranging from 5 to 30% in physiological or pathological conditions, respectively) when grown on type I collagen or fibronectin remain in a quiescent fibroblastic state, whereas those cultured on fibrin surfaces exhibit a myofibroblast phenotype and rapidly form calcified aggregates (Chen and Simmons, 2011). These data further highlight the complex interactions between cells and between cells and matrix.

Therefore, beside alterations in the balance between pro- and anti-calcifying factors, changes in the extracellular matrix may significantly contribute to mineral deposition. It is noteworthy to mention that in soft connective tissues, if mineralization is not triggered by necrotic cell debris, elastic fibers seem to represent the selected target of pathologic mineralization, possibly due to their low turnover and/or susceptibility to calcium ion-binding (Pugashetti et al., 2011).

Purified elastin has been demonstrated to have calcium-binding capabilities (Molinari-Tosatti et al., 1968; Cox et al., 1975; Long and Urry, 1981). Moreover, addition of elastin peptides to cultured SMC enhances Von Kossa positive calcium precipitates in the phosphate model of *in vitro* calcification (Hosaka et al., 2009). In accordance with these data, elastin degradation due to the up-regulation of matrix metalloproteinase (MMP)2, MMP9, and cathepsin S has been shown to increase arterial calcification in the uremic mice model of ectopic calcification (Pai et al., 2011) and to favor calcification of native heart valves (Perrotta et al., 2011). It has been therefore suggested that peptides or fragments derived from elastin degradation, due to their high hydrophobicity and coacervation properties, may enhance abnormal mineralization (Abatangelo et al., 1975; Long et al., 1975) leading to the formation of abnormal complexes with high calcium-binding capabilities (Bell et al., 1974; Tamburro et al., 1977; Urry et al., 1982). These findings sustain the association between inflammation and ectopic calcification, especially in the vascular compartment (Shao et al., 2010).

Beside elastin itself, elastic fibers are made of several components whose exposure, with age and in pathological conditions, further contributes to the preferential localization of ectopic calcifications on elastin and on elastic fiber-associated molecules. For instance, calcium-binding sites have been found on specific domains in fibrillin I (Handford, 2000), one of the principal elastin-associated proteins. Therefore, not only elastin *per se*, but also elastic fibers, as a whole, could function as nucleation center for calcium precipitation (Starcher and Urry, 1973). Beside fibrillin, proteoglycans (PG), glycosaminoglycans (GAGs), and other glycoproteins present inside elastic fibers could also represent additional calcium-binding sites.

We have repeatedly demonstrated that GAGs are present inside elastic fibers, possibly regulating the mechanical properties and stability of these fibers (Pasquali-Ronchetti et al., 1984; Contri et al., 1985). Changes in the type or ratio of GAGs, as those occurring with age, in the course of pathologic conditions or depending on tissue or on specific physiological requirements (Berenson et al., 1985; Cherchi et al., 1990; Passi et al., 1997; Qu et al., 2011), may influence the characteristics of elastic fibers and of the whole extracellular matrix, as demonstrated for instance in the vasculature where connective tissue molecules follow a gradient depending on the distance from the heart (Madhavan, 1977). Moreover, we have also demonstrated that calcified elastic fibers exhibit peculiar type and localization of PG/GAGs such as heparan sulfate, putting forward the hypothesis that GAGs have a role in elastic fiber homeostasis as well as in the calcification process (Passi et al., 1996; Gheduzzi et al., 2005).

Furthermore, it was shown that, in cartilage, PG/GAGs act as calcium-concentrating agents promoting calcification, but they also behave as inhibitors of hydroxyapatite formation functioning as a cation-exchanging calcium reservoir (Hunter, 1991). Consistently, decorin, a small leucine-rich PG containing one dermatan sulfate or chondroitin sulfate chain, beside its regulatory role on transforming growth factor (TGF)-β activity and collagen fibrillogenesis, binds to hydroxyapatite (Boskey et al., 1997; Rees et al., 2001; Mochida et al., 2009) and colocalizes with areas of calcification in skeletal tissues, in the adventitia of blood vessels, and in the skin (Hocking et al., 1998).

A further link between GAGs and the calcification process is the ability of BMPs to bind to heparin and to induce osteoblast differentiation of mesenchymal cells. Moreover, heparan sulfate and dextran sulfate enhanced BMP2 activity serving as ligands to their signaling receptors on cell membranes (Takada et al., 2003).

## ROLE OF MESENCHYMAL CELLS IN SOFT CONNECTIVE TISSUE CALCIFICATIONS

### PERICYTES

The presence of perivascular cells closely associated with capillaries was described more than 100 years ago, although their origin remained elusive for many decades (Díaz-Flores et al., 1991). Some studies proposed that they may derive from the neural crest, whereas other studies suggested that pericytes derive from smooth muscle cells, fibroblasts, endothelial cells, and bone marrow and that they exhibit a multi-lineage potential being capable of differentiating into a variety of cell types including osteoblasts and chondrocytes, as demonstrated, both *in vitro* and *in vivo* experimental models. On the basis of these observations, it was suggested that pericytes play a role in mediating ectopic calcification (Collett and Canfield, 2005).

There is now good evidence that angiogenesis regulates ectopic calcification in several ways: (i) angiogenic factors are mitogenic for mesenchymal cells and osteoblasts and enhance bone formation *in vivo*; (ii) cytokines as BMP2 and BMP4, released by endothelial cells, induce both the differentiation of osteoprogenitor cells and calcification *in vitro* and *in vivo*, although this effect is context-dependent (Shin et al., 2004); (iii) new vessels serve as a conduit for osteoprogenitor cells that may derive from the circulation or from pericytes themselves. Consistently, the association between angiogenesis and ectopic calcification has been noted in several cases, as in ductal carcinoma *in situ*, in calcifying fibroblastic granuloma, in choroidal osteoma and in the calcifications of the retina.

When cultured in standard growth medium, pericytes undergo a process of growth and differentiation characterized by the formation, within approximately 8 weeks, of large multi-cellular nodules that, similarly to the matrix found in calcified vessels, contain type I collagen, OPN, matrix Gla protein and OC and hydroxyapatite crystals with a calcium to phosphate ratio analogous to that of bone (Doherty and Canfield, 1999; Abedin et al., 2004).

### VASCULAR SMOOTH MUSCLE CELLS

Studies in human lesions and mouse models of arterial calcification as well as *in vitro* calcification models of human and bovine VSMC support the concept that mesenchymal-derived vascular cells participate in mineral deposition by mimicking bone formation, since they exhibit several hallmarks of endochondral ossification (Liu and Shanahan, 2011).

It has been clearly demonstrated that VSMC (1) undergo osteoblastic differentiation with loss of smooth muscle-specific gene expression and gain of osteoblast-like properties, including expression of the osteoblast differentiation factor Cbfa-1; (2) activate the mineralization process in the presence of high concentrations of extracellular phosphate; (3) may require a sodium-dependent phosphate co-transporter function to calcify (Giachelli, 2001; Vattikuti and Towler, 2004). The complexity of VSMC phenotypic changes associated to ectopic calcifications has been recently clearly outlined by a whole-genome expression array approach in uremic rats fed on a high phosphate diet. It was in fact demonstrated that the transition from “muscle-related” to “bone-related” gene expression involved the deregulation of at least 53 genes (Román-García et al., 2010) and the activation of Erk1/2 and Wnt pathways (Lau et al., 2010).

Interestingly, it has been shown that, in appropriate culture conditions, approximately 10–30% of VSMC have the capacity to express osteoblast differentiation markers and to retain this phenotype through *in vitro* passages (Boström et al., 1993). In agreement with these *in vitro* data, a variety of bone matrix proteins and regulatory factors have now been demonstrated in human calcified plaque, including OC, BSP, osteonectin, collagen I, alkaline phosphatase, Msx-2, and Cbfa-1.

### FIBROBLASTS

Many studies performed on VSMC demonstrated that mesenchymal cells, whether locally producing pro- and anti-calcifying factors or being involved in extracellular matrix synthesis and degradation, are involved in the mineralization of soft connective tissues. Never the less, a key question is whether all mesenchymal cells behave similarly, or if differences in their tissue-specific differentiation may be associated to a different susceptibility of connective tissues to mineralize.

**FIGURE 2:**
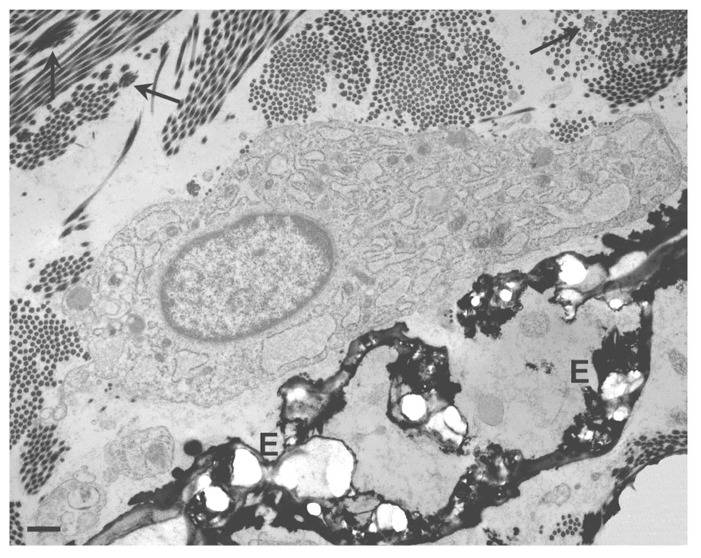
**Transmission electron microscopy of a dermal biopsy from a patient affected by pseudoxanthoma elasticum (PXE)**. Deformed calcified elastic fibers (E) are present in close proximity to large fibroblasts with abundant and dilated cisternae of the endoplasmic reticulum. Collagen fibrils occasionally organized into collagen flowers (arrows) are also visible. Bar = 1 μm.

For instance, skin seems to be only rarely affected by ectopic calcification in contrast to the vascular system. Very few studies have been done on fibroblasts and especially on dermal fibroblasts, although in a number of disorders there is a clear evidence for their close association to ectopic calcifications (**Figure 2**).

Among the few reports on fibroblast-associated calcifications are those showing that a human gingival fibroblast cell line may exhibit both intracellular and extracellular ectopic mineralization starting within round and irregularly shaped vesicles contained in large cytoplasmic vacuoles. This may suggest that mineral accumulation and transformation of amorphous mineral into crystalline structures take place within cellular vesicular structures like MV (Yajima et al., 1984).

By contrast, MV have been never observed within or around dermal fibroblasts in areas of matrix calcification *in vivo* nor in the high phosphate-calcification model *in vitro* (personal observations). This finding indicates the occurrence of different phenotypic characteristics between dermal and gingival fibroblasts, but at the same time demonstrates that mineral deposition can be observed also independently from MV.

In the attempt to understand the interactions between cells from hard and soft connective tissues and to unveil the complexity of the mechanisms involved in fibroblast-associated calcification, Yu and coworkers performed a cDNA microarray analysis on fibroblasts from spinal ligaments cultured in the presence of conditioned media of osteoclast-like cells. In this environment fibroblasts exhibited high levels of alkaline phosphatase and mineral deposition, but more interestingly, microRNA expression profiles revealed a significant down-regulation of a group of microRNAs known to negatively interfere with genes associated with osteogenic differentiation (e.g., BMP2, OC, Runx2). In the light of these data, it has been hypothesized that osteoclasts might induce the osteogenic differentiation of fibroblasts *in vitro* and that miRNA may play an important role in the regulation of cell–cell interactions between osteoclasts and fibroblasts (Yu et al., 2011).

An additional demonstration of the ability of fibroblasts to modulate their phenotype in response to specific environmental characteristics has been provided by studies on rat dermal fibroblasts cultured in the presence of elastin degradation products and of TGF-beta1. Mineralization was preceded by up-regulation of alpha-smooth muscle actin, type I collagen and MMP2, which are characteristic features of myofibroblasts. Thereafter, osteogenic markers as OC, alkaline phosphatase, and osteoprotegerin increased their expression and, after 21 days, multi-cellular calcified nodules were observed. It was proposed that elastin-associated mineralization might result from defective/unbalanced dynamic remodeling events similar to those occurring during the repair process (Simionescu et al., 2007).

Therefore, it is important to note that, irrespective of the cell type, a specific environment is required for calcification to occur, *in vivo*, but especially *in vitro*. All cultured mesenchymal cells, in fact, are dependent for their growth on a variety of cytokines and adhesive molecules as those easily provided by addition of fetal/calf bovine serum. However, the amount of “serum factors” significantly higher compared to physiological “*in vivo*” concentrations, provide cells of a number of other components that, depending on the characteristics of the serum, directly influences the mineralization process or may regulate cell behavior and, as a consequence, the expression of specific gene/proteins. Among these factors, serum fetuin A, that is usually present in cell culture media, represents a powerful inhibitor of the calcification process making cells unable to mineralize in standard cell culture conditions. To overcome this problem, it is possible to utilize serum-free media (i.e., media with chemically defined components and supplements) and/or to add to standard cell cultures [in Dulbecco’s modified Eagle’s medium (DMEM) plus serum] high concentrations of phosphate (that can easily precipitate as soon as it forms complexes with calcium) or of phosphate donor substrates (that require an active involvement of cells for the enzymatic release of inorganic phosphate from substrates). Human skin-derived fibroblast precursor cells, for instance, can acquire an osteoblast-like behavior and start to mineralize the newly deposited extracellular matrix only if cultured in a pro-osteogenic medium (supplemented with ascorbic acid, beta-glycerophosphate, and dexamethasone); as a result induced expression of alkaline phosphatase, BSP and OC leads to mineral deposition (Buranasinsup et al., 2006).

**FIGURE 3:**
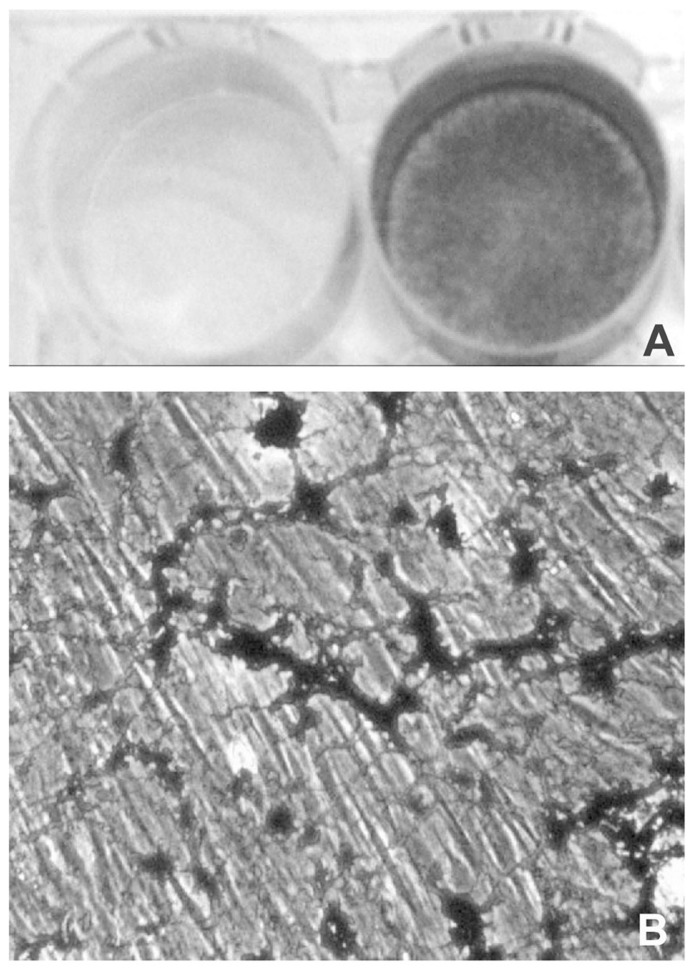
**Light microscopy of dermal fibroblasts cultured for 3 weeks in the presence of DMEM (A) or of DMEM supplemented with ascorbate, beta-glycerophosphate, and dexamethasone (B)**. In the presence of the calcified medium, dermal fibroblasts exhibit areas of mineralization that can be clearly visualized upon Von Kossa staining as dark deposits (lower panel; see also Boraldi et al., in press, for methodological details).

A further difference, between mesenchymal cell types is the time required *in vitro* to obtain a calcified matrix, which may be taken as predictive of the pro-osteoblastic potential of the cells. In osteoblast and VSMC cultures, mineralization can be obtained after 3–5 or 8–10 days in culture, respectively (Uchimura et al., 2003; Li et al., 2004). By contrast, mineralized matrix becomes clearly evident only after 2–3 weeks in dermal fibroblast cultures (Boraldi et al., in press; **Figure 3**).

On the basis of these results it could be hypothesized that dermal fibroblasts are rather resistant to be converted into osteoblast-like cells, in agreement with the uncommon occurrence of dermal calcifications.

Interestingly, ectopic soft tissue calcification is a well-known symptom in Werner syndrome (WS), an autosomal recessive progeroid disorder caused by mutations in RecQ DNA helicase. Cultured fibroblasts from WS patients undergo spontaneous mineralization *in vitro* at normal phosphate concentration, and overexpress Pit-1 at mRNA and protein levels. Both calcification and Pit-1 up-regulation have been also detected *in situ* in the skin of patients (Honjo et al., 2008), supporting the concept that dermal fibroblasts mimic and retain *in vitro* at least some of the pathologic characteristics they have *in vivo*, thus representing a valuable model to investigate the pathogenetic mechanisms of diseases.

## PXE AND PXE-LIKE DISORDERS AS MODELS FOR INVESTIGATING THE ROLE OF FIBROBLASTS IN SOFT CONNECTIVE TISSUE CALCIFICATIONS

### PSEUDOXANTHOMA ELASTICUM

Pseudoxanthoma elasticum is a rare genetic disorder characterized by skin papules on the neck, axillae, and groin, often associated with skin redundancy and laxity, by retinal alterations as angioid streaks and neovascularization, and by middle sized artery narrowing up to occlusion. All these alterations depend on the deposition of calcium minerals inside or associated with elastic fibers (Truter et al., 1996; Gheduzzi et al., 2003; **Figure 2**). The phenomenon is rather peculiar as calcifications affect only elastic fibers, whereas collagen does not calcify; moreover, this abnormal mineralization occurs in the absence of increased calcium and phosphate levels, in the total absence of inflammation, cell necrosis, apoptosis.

Pseudoxanthoma elasticum has been associated to mutations in the *ABCC6* gene, a member of the ABC family of membrane transporters (it encodes for the membrane transporter MRP6), that is mainly expressed in liver and kidney (Bergen et al., 2000; Le Saux et al., 2000; Ringpfeil et al., 2000), whereas its expression is surprisingly low in tissues specifically involved in the clinical manifestations of PXE. In this context, it has been suggested that a still unknown circulating metabolite released (or not released) by the liver in *ABCC6* deficiency may directly affect elastic fiber formation, stability, and calcification (Le Saux et al., 2006).

Actually, in a setting of normal calcemia and phosphatemia, several abnormalities have been documented in the circulation of PXE patients, from PGs and enzymes involved in their synthesis (Götting et al., 2005; Schön et al., 2006; Volpi and Maccari, 2006), to protein and lipid abnormalities indices of oxidative stress (Garcia-Fernandez et al., 2008), to high levels of MMP2 and MMP9 (Diekmann et al., 2009) and of elastin-derived peptides (Annovazzi et al., 2004; **Table 1**).

**Table 1:** Genes/molecules involved in the regulation of elastic fiber calcification in PXE.

Regulators of mineral formation/deposition	PXE findings (reference)
Circulating ion levels	Normal Ca and P (Boraldi et al., in press)
Phosphate homeostasis	Normal serum alkaline phosphatase (ALP; Boraldi et al., in press) ↑ TNAP in fibroblasts (Boraldi et al., in press)
Calcification inhibitors	↓ Gla-MGP (Gheduzzi et al., 2007; Li et al., 2007; Hendig et al., 2008b) ↓ Fetuin-A (Hendig et al., 2006; Jiang et al., 2010) Polymorphisms of OPN (Hendig et al., 2007) Mutation of ENPP1 (Nitschke et al., 2012)
Extracellular matrix components	↑ MMP2, MMP9 in serum (Diekmann et al., 2009) ↑ MMP2 in fibroblasts (Quaglino et al., 2005) Polymorphisms of MMP2 (Zarbock et al., 2010) ↑ Elastin-derived peptides (Annovazzi et al., 2004) Different ratio PG/GAGs (Tiozzo-Costa et al., 1988; Passi et al., 1996; Maccari and Volpi, 2008) ↑ Circulating selectins (Götting et al., 2008) ↑ Serum intercellular adhesion molecule (ICAM; Hendig et al., 2008a) Different expression of integrins (Quaglino et al., 2000) ↑ Serum XT-I activities (Götting et al., 2005) Polymorphisms of XT-I (Schön et al., 2006)
Redox balance	↑ ROS in serum (Garcia-Fernandez et al., 2008) ↑ ROS in fibroblasts (Pasquali-Ronchetti et al., 2006; Boraldi et al., 2009) Polymorphisms of antioxidant genes (Zarbock et al., 2007)

Moreover, low levels of fetuin A and of vitamin K have been measured in the circulation of PXE patients and in the PXE animal model (Jiang et al., 2010; Vanakker et al., 2010). Low levels of fetuin A could be explained by the augmented capture of this molecule by peripheral mineral precipitates (Price et al., 2004; Hendig et al., 2006), although it cannot be excluded that PXE fibroblasts may sequester this inhibitor (Boraldi et al., 2007) as a consequence of higher intracellular uptake that may prevent fetuin A from exerting its regulatory role in peripheral tissues. The recent finding that in the mouse model of PXE ectopic calcification can be significantly reduced by overexpressing fetuin A (Jiang et al., 2010) may suggest that in PXE the role of this inhibitor should be further investigated.

Nevertheless, it is unlikely that all these plasma abnormalities in PXE patients would directly depend on the deficiency of the membrane transporter MRP6. It would seems more reasonable that inherited *ABCC6* deficiency, along the years, would induce a series of metabolic adjustments in several tissues possibly epigenetically involving a network of different genes and leading to the complexity and heterogeneity of PXE alterations. Moreover, since each patient has a different genetic background, the consequences of these “metabolic adjustments” would be different in each individual thus explaining the extreme variability of clinical manifestations among patients.

Therefore, clinical and experimental data strongly suggest that elastic fiber calcification in PXE is not a passive process merely due to the presence or absence of one or more abnormal plasma components, but the result of activities mediated by local cells. At least in skin, fibroblasts should be considered the principal candidates for several reasons.

First of all, if elastic fiber calcifications in PXE are a passive process due to the infiltration of plasma molecule(s), all elastic fibers should calcify; on the contrary, skin elastic fiber calcification is present only in peculiar regions of the body. Moreover, also in areas prone to calcification, not all elastic fibers mineralize. This is in agreement with the overgrowing evidence of the diversity of skin fibroblasts at different anatomical sites, as these cells display distinct and characteristic transcriptional patterns for a large number of genes depending on the body region they come from (Chang et al., 2002; Lindner et al., 2012). Therefore, skin fibroblasts may be considered differentiated cell types that, depending on their location, maintain their positional identities even when isolated and cultured *in vitro* (Rinn et al., 2008) and probably react in different ways to abnormal exogenous stimula, such as those present in the circulation of PXE patients.

A second evidence for the involvement of fibroblasts in skin abnormalities in PXE is, beside elastic fiber calcification, the documented presence of huge aggregates of PGs and of various extracellular matrix proteins in the affected areas of the dermis (Pasquali-Ronchetti et al., 1986; Tiozzo-Costa et al., 1988; Baccarani-Contri et al., 1994; Passi et al., 1996), consistent with a significant increase of the total amount of GAGs in the skin of patients (Maccari and Volpi, 2008; **Table 1**) and with the observed decreased susceptibility of GAG-associated elastin to pancreatic elastase (Schwartz et al., 1991). Such structural and chemical alterations, very likely responsible for skin redundancy and laxity in the affected areas, must be under the local control of fibroblasts, which are responsible for the synthesis of the extracellular milieu in soft connective tissues.

An indirect indication that also in the vessel wall fibroblasts are probably involved in the early calcification of elastic fibers is the observation that calcification in PXE vessels is often present within the elastic fibers close to the adventitia, in the absence of any osteoblast-like phenotype of the adjacent cells, that in fact maintain a fibroblast-like appearance (Gheduzzi et al., 2003).

Finally, several studies by our and other groups have shown that fibroblasts isolated from the dermis of PXE patients have and maintain *in vitro* a metabolic behavior different from fibroblasts isolated from the same body areas of gender and age-matched normal subjects. Actually, it has been demonstrated that PXE fibroblasts suffer from an oxidative stress condition (Pasquali-Ronchetti et al., 2006), produce highly sulfated GAGs (Tiozzo-Costa et al., 1988; Passi et al., 1996), exhibit abnormal proteoglycanase (Gordon et al., 1978, 1983) and higher metalloproteinase activities (Quaglino et al., 2005), are unable to properly carboxylate MGP (Gheduzzi et al., 2007; Boraldi et al., in press; **Table 1**), and have a different protein profile (Boraldi et al., 2009) indicating that their metabolic behavior is genetically or epigenetically different from control fibroblasts and is maintained when cells are cultured *in vitro* (Rinn et al., 2008).

In the light of these observations, dermal fibroblasts from PXE patients can be considered a valuable and very informative model to better understand the contribution of these cells to soft connective tissue calcifications. Therefore, beside the role of fibroblasts in regulating the characteristics of the extracellular environment and, as a consequence, the different susceptibility of tissues and of elastic fibers to calcify, a key question remains whether the same osteoblast-related pathways, as those described in SMC, are also involved in the phenotype of fibroblasts prone to calcify (i.e., PXE fibroblasts).

Recent evidence from our laboratory indicates that TNAP activity, although within normal range in the circulation of patients, is higher in PXE fibroblasts compared to control cells and that these differences are further amplified when cells are grown in a calcifying medium. The process is rather complex, however, data suggested that the local increase of phosphorus in the extracellular milieu together with the reduced amount of anti-calcific molecules, such as carboxylated MGP, may favor hydroxyapatite formation (Boraldi et al., in press).

Interestingly, low levels of MGP have been found in the circulation of PXE patients (Hendig et al., 2008b) and in the *Abcc6*-/- mice model of PXE (Li et al., 2007). In accordance, low levels of carboxylated MGP are produced by skin PXE fibroblasts *in vitro*, indicating that the local synthesis of the mature protein is of paramount importance in preventing elastic fiber calcification (Gheduzzi et al., 2007) and that fibroblasts are likely involved in the local secretion of this important anti-calcific protein. Since MGP γ-carboxylation is a vitamin K-dependent process (Theuwissen et al., 2012), it was suggested that PXE calcification could be due to vitamin K deficiency (Borst et al., 2008). Although the level of vitamin K is low in PXE patients (Vanakker et al., 2010), nevertheless the availability and the cellular utilization of vitamin K do not seem responsible for MGP under-carboxylation. Both in PXE fibroblasts and in two different *Abcc6*-/- mice models, addition of vitamin K did not improve MGP carboxylation (Boraldi et al., in press) nor prevented calcification in spite of the high serum concentration of vitamin K upon treatments (Brampton et al., 2011; Gorgels et al., 2011; Jiang et al., 2011). Therefore, low vitamin K does not seem to play a pivotal role in MGP carboxylation nor in elastic fiber calcification in PXE. Moreover, in PXE, carboxylation of proteins involved in coagulation or in bone calcification seems adequate, as no defects in coagulation or in bone mineralization have been described in patients. Therefore, both vitamin K availability and the carboxylase system do not seem directly involved in PXE mineralization. Recent data from our laboratory seem to suggest that the low carboxylation rate of MGP by PXE skin fibroblasts, even in a setting of high vitamin K concentration, might depend on the intrinsic ability of MGP to be carboxylated (Boraldi et al., in press)

In addition, evidence has been provided through the years that the PXE phenotype can be obtained through pathways other than those caused by *ABCC6* mutations. An indirect proof of this is that PXE-like clinical and histo-pathological manifestations have been described in a number of patients affected by beta-thalassemia (Aessopos et al., 1992, 1998; Baccarani-Contri et al., 2001; Cianciulli et al., 2002) and other hemoglobinopathies (Goldberg et al., 1971; Nagpal et al., 1976; Aessopos et al., 2002; Fabbri et al., 2009), in subjects treated with penicillamine (Rath et al., 2005), in cases of γ-carboxylase gene (*GGCX*) and *ENPP1* mutations (Vanakker et al., 2007; Le Boulanger et al., 2010; Li et al., 2012; Nitschke et al., 2012; see further for additional data) and, more recently, in a few cases of liver transplantation where the impossibility to examine the DNA from all donors and recipients made not clear if the transplanted liver harbored or not *ABCC6* mutations (Bercovitch et al., 2011).

### PXE-LIKE DISORDERS

#### Beta-thalassemia

As already mentioned, the metabolic complexity at the basis of elastic fiber calcification could, at least partially, explain the phenotypic similarities of the skin lesions in inherited PXE and in a number of different unrelated disorders, such as in patients affected by beta-thalassemia (Aessopos et al., 1992; Baccarani-Contri et al., 2001).

More than 60 years ago, elastinopathies similar to that in PXE were documented in sickle cell anemia (Paton, 1959; Suerig and Siefert, 1964) and, later, in a series of hemoglobinopathies (Nagpal et al., 1976), among which β-thalassemia (Aessopos et al., 1992, 1997). Subsequent studies better defined the almost identical clinical and histo-pathological alterations in PXE and in a relevant number of β-thalassemia patients (Aessopos et al., 1998). In both these unrelated genetic disorders, angioid streaks (Gibson et al., 1983; Kinsella and Mooney, 1988; Aessopos et al., 1989, 1992; O’Donnell et al., 1991), arterial elastorrhexis, and calcification (Aessopos et al., 1998; Tsomi et al., 2001; Cianciulli et al., 2002) as well as coalescent skin papules on the posterior/lateral aspect of neck, axillae, and groin with elastic fiber calcification (Aessopos et al., 1992; Baccarani-Contri et al., 2001) have been repeatedly described.

Skin abnormalities in genetic PXE and in beta-thalassemia patients with clinical PXE-like manifestations (β-thal/PXE) have been carefully analyzed. It was observed that both disorders had identical elastic fiber calcifications, “collagen flowers” abnormalities, as well as cell and matrix alterations, suggesting that similar metabolic changes could be involved in both disorders as final consequence of mutations in apparently unrelated genes.

In agreement with this hypothesis are data from experiments aiming to verify if the similarities of clinical and histo-pathological features in genetic PXE and in β-thal/PXE patients could be sustained by analogous similarities in the metabolic behavior of cultured fibroblasts.

**FIGURE 4:**
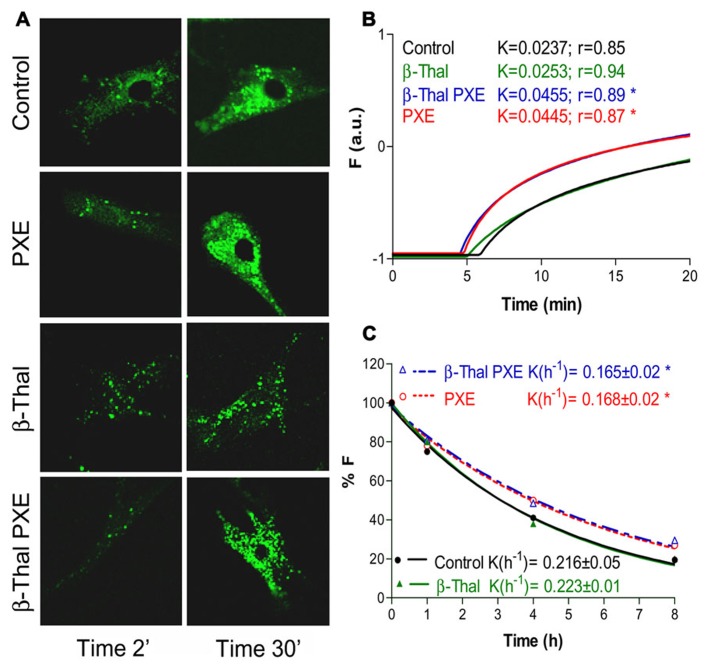
**Calcein uptake and extrusion in cultured fibroblasts from healthy individuals (Control), PXE patients (PXE), beta-Thalassemic patients with (βThal/PXE) and without (βThal) PXE-like manifestations**. Intracellular calcein accumulation is shown after 2 and 30 min by confocal microscopy **(A)**. Calcein uptake **(B)** and extrusion **(C)** have been measured by flow cytometry and are shown in panels on the right (see also Boraldi et al., 2003, for methodological details).

Fibroblasts from healthy subjects, from PXE patients as well as from individuals affected by beta-thalassemia exhibiting (β-thal/PXE) or not (β-thal) ectopic calcification, were investigated for their ability to accumulate and to extrude calcein-AM (acetomethoxy derivate of calcein; Boraldi et al., 2008), a chemical widely used for determining cell viability. The non-fluorescent calcein-AM enters living cells where it is hydrolyzed by intracellular esterases into the strongly fluorescent green anion calcein that can be retained in the cytoplasm or actively extruded. The accumulation and the extrusion of fluorescent calcein can be easily visualized by confocal microscopy and quantified by flow cytometry (Boraldi et al., 2003). Within few minutes of incubation with calcein-AM (0.1 μM) dermal fibroblasts accumulate the fluorescent molecule into discrete granules in the cytoplasm. After a 30-minute incubation, calcein accumulation is much higher in PXE and in β-thal/PXE cells than in controls and in β-thal fibroblasts. Furthermore, also calcein efflux from β-thal/PXE fibroblasts is significantly different compared to controls (p < 0.05), whereas it is identical to that of fibroblasts from patients with inherited PXE (**Figure 4**). Therefore, *in vitro* dermal fibroblasts from β-thal/PXE patients, in the absence of *ABCC6* mutations (Hamlin et al., 2003), exhibit functional alterations similar to those of fibroblasts isolated from patients with inherited PXE. In this specific case, the calcein assay is defective in fibroblasts isolated from subjects with identical elastic fiber calcification (i.e., PXE and β-thal/PXE), whereas it is normal in fibroblasts from β-thalassemia patients without PXE-like clinical alterations. Therefore, these findings further support the hypothesis that PXE-like clinical manifestations described in some β-thalassemia patients might derive from metabolic alterations occurring in this particular sub-group of patients and that similar pathways may be at the basis of elastic fiber calcification in inherited PXE and in β-thal/PXE patients (Boraldi et al., 2008).

In β-thalassemia patients an abnormal oxidative stress induced by the iron overload derived from repeated transfusions, by unpaired alpha-globin chains (Livrea et al., 1996; Hershko et al., 1998; Cighetti et al., 2002) and by deficient oxygen transport to peripheral tissues (Chan et al., 1999; Meral et al., 2000) has been described. How oxidative stress interferes with connective tissue metabolism is still largely unknown. It has been shown that iron overload and the consequent oxidative stress affect the synthesis of elastin by human dermal fibroblasts *in vitro* (Bunda et al., 2005) and that genetic factors as well as environmental oxidative stress may deeply influence the extracellular matrix and the behavior of cells *in vivo* (Hanley and Repine, 1993). It could be suggested that clinical and histological alterations in β-thal/PXE patients could be due, at least in part, to chronic oxidative stress that, similarly to inherited PXE (Garcia-Fernandez et al., 2008), is not adequately compensated due to a peculiar genetic/epigenetic background. Interestingly, the introduction of oral iron chelators markedly increased the survival of β-thalassemia patients (Borgna-Pignatti et al., 2004) and reduced the level of oxidative stress, in agreement with the observation that PXE-like clinical manifestations are never found in properly treated new β-thalassemia patients (personal observation).

Interestingly, *Hbb^th3/+^* mice are characterized by a significant liver-specific decrease of mrp6 production, due to failure of the NF-E2p45 transcription factor to bind to the *Abcc6* proximal promoter. Even though this animal model of thalassemia is not characterized by soft connective tissue mineralization, never the less it demonstrates that *Abcc6* gene expression can be modified by environmentally-induced changes in transcription factor activity (Martin et al., 2011) and that oxidative stress could play a relevant role. In this context, it is worthwhile to mention that there are data in the literature in favor of a relationship between NF-E2p45 and Nrf2 transcription factors, as independent groups have shown the role hemin in stimulating the expression of antioxidant heme oxygenase 1 (Li et al., 2011) as well as in inducing beta-globin gene expression through the functional intervention of p45NF-E2 transcription factor (Moore et al., 2006). Moreover, in favor of a negative effect of oxidative stress on the expression of *ABCC6* are data showing that vitamin K3 and oxidant agents induce down-regulation of *ABCC6* expression in HepG2 cells (De Boussac et al., 2010).

#### Deficit of vitamin K-dependent gamma-carboxylase system

The vitamin K-dependent gamma-carboxylation system is composed of the gamma-carboxylase and the warfarin-sensitive enzyme vitamin K(1) 2,3-epoxide reductase (VKOR), which are located in the endoplasmic reticulum where they interact with other proteins like calumenin and protein disulfide isomerase, negative and positive regulators of the vitamin K cycle, respectively (Wajih et al., 2004; Wallin et al., 2008). Different expression of these two regulatory proteins has been demonstrated on *in vitro* cultured fibroblasts to be probably involved in the pathogenesis of PXE and of PXE-like calcifications (Boraldi et al., 2009).

During vitamin K-dependent post-translational gamma-glutamyl carboxylation, vitamin K hydroquinone is oxidized to the epoxide form (K>O) that, in turn, is reduced by the enzyme VKORC1 (vitamin K epoxide reductase complex component 1) to complete the vitamin K cycle.

The demonstration that the enzyme VKORC1 is the target for the anti-coagulant drug warfarin and that patients treated with this drug develop extensive vascular calcifications (Palaniswamy et al., 2011) sustain the importance of the vitamin K-dependent regulatory mechanisms of calcification. In particular, VKORC1 appears to be a rate-limiting step in the biosynthesis of functional vitamin K-dependent proteins.

Interestingly, skin lesions due to elastic fiber calcification almost identical to those in PXE have been described in cases of mutations of *GGCX* (Vanakker et al., 2010). As already mentioned, this enzyme is necessary for the γ-carboxylation of a number of proteins some of which are involved in ectopic calcification (Shanahan et al., 1998; Price et al., 2006). Therefore, mutations in the *GGCX* gene are at the basis of an autosomal recessive disorder characterized by a generalized deficiency of the Vitamin K-dependent clotting factors as well as mineralization and fragmentation of elastic fibers leading to thickened, inelastic skin and limited retinopathy, associated to accumulation of uncarboxylated Gla proteins (MGP and OC) in plasma, serum and dermis, in the presence of normal serum levels of vitamin K (McMillan and Roberts, 1966). Even though, the deficient carboxylation of coagulation proteins can be restored by vitamin K administration that increases the level of the electron-donor hydroquinone form of vitamin K available for GGCX, never the less, 1 year treatment with vitamin K did not ameliorate skin lesions nor elastic fiber calcification in one patient affected by *GGCX* mutations (unpublished observations). It could be suggested that carboxylase is essential for maturation of MGP, but that the electron donor level of vitamin K does not influence the performance of MGP carboxylation. These data and those from other laboratories showing that vitamin K supplementation does not increase the level of circulating carboxylated MGP in a case of Keutel syndrome (*MGP* mutations; Cranenburg et al., 2011) seem to indicate that MGP carboxylation is under a complex control, only partly dependent on vitamin K, in agreement with recent results obtained on PXE fibroblasts treated *in vitro* with vitamin K supplementation (Boraldi et al., in press).

To further enlarge the spectrum of ectopic calcification disorders which are clinically and/or pathogenetically related to PXE, there is a recent report describing a patient, bearing two *ABCC6* mutations and a gain of function single-nucleotide polymorphism (SNP) in the *GGCX* gene, who was characterized by both classic PXE (papules, retinopathy, and calcifications) and by a PXE-like syndrome (cutis laxa beyond the flexural areas; Vanakker et al., 2011).

Mutations in the *GGCX* or *VKORC1* genes are associated with a hereditary deficiency of the vitamin K-dependent clotting factors as well as a clinically relevant dependency of anti-coagulants (Brenner, 2000; Vanakker et al., 2010). Besides these enzymatic defects, a deficiency of vitamin K has been described in association with coagulation, bone (osteoporosis, osteoarthritis) and vascular (arteriosclerosis) disorders resulting from insufficient carboxylation of Gla proteins (Neogi et al., 2006).

#### Generalized arterial calcification of infancy

Generalized arterial calcification of infancy is associated with mutations in the *ENPP1* gene and is characterized by mineralization of the internal elastic lamina of large and medium-sized arteries and stenosis due to myointimal proliferation. Although survival to adulthood has been reported, most patients die within the first 6 months of life (Rutsch et al., 2003).

Features of PXE have been recently described in patients with homozygous missense mutation of the *ENPP1* gene (Li et al., 2012). Cutaneous calcification was never been previously described in ENPP1 deficiency and this finding is a clear demonstration of the role of PPi as a critical anti-calcific agent in PXE and PXE-like disorders.

It is therefore noteworthy the occurrence of a clinical and genetic overlapping between PXE and GACI as clearly highlighted by a recent study on two brothers born from to unrelated parents, showing that the elder developed a PXE condition bearing *ABCC6* mutations, whereas the younger died at 15 months of age of a condition clinically reminiscent of GACI, although it appeared independent of *ENPP1* mutations (Le Boulanger et al., 2010).

The finding that MGP and fetuin A are involved in both conditions further sustain the hypothesis that *ABCC6* mutations account for a significant subset of GACI patients, and *ENPP1* mutations can also be associated with PXE lesions in young children, thus reflecting two ends of a clinical spectrum of ectopic calcifications, possibly through the involvement of common physiological pathways (Nitschke et al., 2012).

## CONCLUSION

In spite of the extreme complexity and still incomplete knowledge of the various actors involved, we have reported evidence supporting the importance of mesenchymal cells, and of fibroblasts in particular, in the occurrence and development of soft connective tissue calcifications. Within this context, fibroblasts from PXE and PXE-like disorders offer a valuable model to better understand the complex pathways that end up with elastic fiber mineralization. It can be argued that not all mesenchymal cells behave in the same way and that morpho-functional characteristics of tissues as well as composition of the extracellular matrix and exogenous agents should be taken into account for understanding the susceptibility/resistance to calcification of different body regions in physiological conditions, in aging and in both genetic and acquired disorders.

## Conflict of Interest Statement

The authors declare that the research was conducted in the absence of any commercial or financial relationships that could be construed as a potential conflict of interest.
